# Clade II *Candida auris* possess genomic structural variations related to an ancestral strain

**DOI:** 10.1371/journal.pone.0223433

**Published:** 2019-10-09

**Authors:** Tsuyoshi Sekizuka, Shigekazu Iguchi, Takashi Umeyama, Yuba Inamine, Koichi Makimura, Makoto Kuroda, Yoshitsugu Miyazaki, Ken Kikuchi

**Affiliations:** 1 Pathogen Genomics Center, National Institute of Infectious Diseases, Tokyo, Japan; 2 Department of Infectious Diseases, Tokyo Women’s Medical University, Tokyo, Japan; 3 Department of Chemotherapy and Mycoses, National Institute of Infectious Diseases, Tokyo, Japan; 4 Department of Medical Mycology, Graduate School of Medicine, Teikyo University, Tokyo, Japan; Vallabhbhai Patel Chest Institute, INDIA

## Abstract

*Candida auris* is an invasive and multidrug-resistant ascomycetous yeast that is under global surveillance. All clinical cases of *C*. *auris* infection diagnosed from 1997 to 2019 in Japan were non-invasive and sporadic otitis media cases. In the present study, we performed whole-genome sequencing of seven *C*. *auris* strains isolated from patients with otitis media in Japan, all of which belonged to clade II. Comparative genome analysis using the high-quality draft genome sequences JCM 15448^T^ revealed that single nucleotide variations (SNVs), clade-specific accessory genes, and copy number variations (CNVs) were identified in each *C*. *auris* clade. A total of 61 genes involved in cell wall and stress response-related functions was absent in clade II, and the pattern of conserved CNVs in each clade was more stable in clade II than in other clades. Our data suggest that the genomic structural diversity is stable in *C*. *auris* isolated from each biogeographic location, and Japanese strains isolated from patients with otitis media might belong to an ancestral type of *C*. *auris*. One Japanese strain, TWCC 58362, with reduced susceptibility to fluconazole, exhibited no mutation in ergosterol biosynthesis-related genes (ERG). However, TWCC 58362-specific variations, including SNVs, indels, and CNVs were detected, suggesting that gene duplication events in *C*. *auris* might contribute to antifungal drug resistance. Taken together, we demonstrated that genomic structural variations in *C*. *auris* could correlate to geographical dissemination, epidemiology, lesions in the host, and antifungal resistance.

## Introduction

*Candida albicans* is one of the most pathogenic ascomycetous yeasts and remains the most common cause of *Candida* bloodstream infections (BSI). As with other pathogenic *Candida* species, the Center for Disease Control and Prevention (CDC) has alerted the global emergence of invasive infections caused by multidrug-resistant *C*. *auris* since 2016 (https://www.cdc.gov/fungal/candida-auris/candida-auris-alert.html), with large-scale nosocomial invasive infection cases caused by *C*. *auris* from at least five continents [1–12].

Although *C*. *auris* is associated with a high mortality rate, treatment is limited because of resistance to antifungal drugs [13]. It is reported that 93% and 35% of *C*. *auris* isolates were resistant to fluconazole (FLC) and amphotericin B, respectively, and that mutations in *ERG11* and biofilm formation were associated with FLC resistance [13,14]. To make matters worse, *C*. *auris* can survive environmental cleaning with chorine-based reagents and hydrogen peroxide vapor [12]. Previous reports indicate that *C*. *auris* in nosocomial cases was transmitted by reusable axillary temperature probes, and could persist in health care settings, suggesting that reusable patient equipment might serve as a source of outbreaks of infection with *C*. *auris* [15]. Thus, monitoring of *C*. *auris* for the prevention of outbreaks should take place in both patients and in the environment.

In Japan, two large-scale fungal infection surveys have been conducted: (i) 1,486 clinical strains were isolated from clinical specimens from 2001 to 2005 [16]; and (ii) 316 strains were isolated from candidemia from 2002 to 2013 [17]. In total, 1,787 strains were identified, and *C*. *auris* was first isolated from the external ear canal of a patient in Japan in 2005 [18]. The second Japanese *C*. *auris* strain was identified from aural discharge [19]. Intriguingly, these two Japanese *C*. *auris* strains were isolated from patients with non-invasive sporadic cases of otitis media. In 2009, *C*. *auris* was also identified in patients with chronic otitis media in South Korea [20]. Kwon *et al*. described 57 clinical isolates of *C*. *auris* isolated from the ear obtained from 13 hospitals in Korea [21], with BSI caused by *C*. *auris* reported in South Korea in 1996, 2009, and 2017. All Korean isolates in these cases belonged to clade II [1,20,21]. Although Japan and South Korea are relatively close geographically, invasive infections caused by *C*. *auris* have not been reported in Japan as of 2019 [22]. The two previously reported Japanese *C*. *auris* strains were susceptible to antifungal agents [18,19]. The Japanese strains might therefore differ significantly from those isolated in other countries, which are resistant to one or multiple antifungal drugs and are highly pathogenic.

Phylogenomic analysis of *C*. *auris* isolates from several countries has been previously reported [13,15,23,24], indicating that the genotypic clade of *C*. *auris* is closely related to its geographical location, such as Venezuela, Japan, South Africa, India, and Pakistan. Thus far, the available genomic sequences of *C*. *auris* in the NCBI database were obtained from mainly nosocomial invasive infection cases in Australia, Colombia, Germany, India, Iran, the Netherlands, Pakistan, Russia, South Africa, the United Kingdom, the United States and Venezuela. One genomic sequence of a Japanese isolate (strain B11220) from a sporadic case was reported, possibly because of the lack of outbreak cases in Japan [25]. However, the phylogeny of the Japanese isolate is clearly unique compared to that of isolates from other countries.

In the present study, we aimed to reveal the unique characters of *C*. *auris* strains isolated in Japan. We obtained the whole genome sequence of seven *C*. *auris* strains isolated from otitis media patients in Japan and constructed a high-quality draft genome sequence of the first reported isolate, type strain JCM 15448^T^. Moreover, we performed phylogenomic and comparative genomic analysis among Japanese and global strains, to reveal invasive multidrug-resistant features of different *C*. *auris* strains. These results contribute to the global effort to manage *C*. *auris* infections, particularly in drug-resistant strains.

## Results

### Isolation and antifungal susceptibility of *C*. *auris* strains obtained from patients in Japan

In Japan, the first and second cases of *C*. *auris* infection were in 2005 and 2017, respectively [18,19] (Table 1). From 1997 to 2008, five ascomycetous yeast strains, which could not be classified using a conventional fungal identification kit, were isolated from patients with otitis media in Japan. The classification of these strains indicated that all strains belong to *C*. *auris* (Table 1). Two of these five isolates, TWCC 13846 and TWCC 13847, were isolated from the left and right ear of the same patient with otitis media on same date, respectively. An antifungal susceptibility test revealed that one of the seven strains, TWCC 58362, was not susceptible to FLC (Table 1).

**Table 1:** Metadata information and biological characterization of *Candida auris* strains isolated in Japan.

Strain name	JCM 15448^T^	TWCC 13846	TWCC 13847	TWCC 13878	TWCC 50952	TWCC 58191	TWCC 58362
Country	Japan	Japan	Japan	Japan	Japan	Japan	Japan
Prefecture	Tokyo	Tokyo	Tokyo	Tokyo	Iwate	Tokyo	Tokyo
Year	2005	2005	2005	1997	2008	2017	2008
Host	Human	Human	Human	Human	Human	Human	Human
Disease	Otitis media	Otitis media	Otitis media	Otitis media	Otitis media	Otitis media	Otitis media
Source	Auditory canal	Otorrhea	Otorrhea	Otorrhea	Otorrhea	Otorrhea	Otorrhea
MIC (mg/L)							
Micafungin	0.03	0.03	0.03	0.06	0.03	0.06	0.125
Caspofungin	N.A.	0.25	0.25	0.5	0.25	0.5	0.125
Amphotericin B	N.A.	0.25	0.25	0.25	≤0.03	0.25	0.25
5-Fluorocytosine	≤0.031	≤0.125	≤0.125	0.5	≤0.125	0.25	0.5
Fluconazole	1	4	1	16	2	4	>64
Itraconazole	≤0.015	0.03	≤0.015	0.125	0.03	0.06	0.25
Voriconazole	≤0.015	≤0.015	≤0.015	0.125	0.03	0.03	2
Miconazole	N.A.	≤0.03	≤0.03	0.06	≤0.03	0.125	1
Reference (PubMed ID)	19161556	This study	This study	This study	This study	29491246	This study
BioSample ID	SAMD00117055	SAMD00117056	SAMD00117057	SAMD00117058	SAMD00117059	SAMD00117060	SAMD00117061
Run ID	DRR129819, DRR129826	DRR129820	DRR129821	DRR129822	DRR129823	DRR129824	DRR129825
Whole genome sequencing	NextSeq, Sequel	MiSeq	MiSeq	MiSeq	MiSeq	MiSeq	MiSeq
ST	2	2	2	2	2	2	2
Clade	II	II	II	II	II	II	II

N.A., Not applicable; ST, sequence type; orange shading, not susceptible.

### High-quality draft-chromosomal and complete mitochondrial DNA sequences of *C*. *auris* JCM 15448

The draft-genome sequencing of *C*. *auris* JCM 15448 was obtained using a long-read library in PacBio Sequel, followed by error correction with paired-end short-read libraries using NextSeq. In addition, *de novo* assembly with only short reads was also performed. The statistics of NGS read and assembly data is summarized in S1 Fig. Twelve contigs were confirmed by circularization, with eleven and one contigs representing the draft chromosomal linear DNA and complete mitochondrial circular DNA (mtDNA), respectively (Table 2). The total contig size of the chromosomal and mtDNA were 12,098,315 and 27,071 bp, respectively, and the number of predicted genes (CDS, rRNA, and tRNA) are shown in Table 2. The total genome length of *C*. *auris* JCM 15448 is 229–615 kb shorter than those of *C*. *auris* B8441, B11221, and B11243 and is almost the same as that of *C*. *auris* B11220 (S1 Table). The average read depth of contig_7 and mtDNA was higher than that of the other contigs (Table 2). The nucleotide sequence structure of contig_7 includes 4 tandem repeats of an rRNA gene cluster (18S, 5.8S, 25S, and 5S ribosomal RNA gene). Contig_7 has 4 times the average coverage ratio against chromosome coverage, suggesting that *C*. *auris* JCM15448 possesses more than 4 rRNA gene clusters. The copy number of mtDNA is approximately 9 copies (Table 2). The comparison of mtDNA sequences among *C*. *albicans* SC5314, *Clavispora lusitaniae* CBS 6936, *C*. *auris* JCM 15448, *C*. *duobushaemulonii* B09383, and *C*. *haemulonii* B11899 suggests a diversity of synteny and structure in *Saccharomycetales*, and the existence of introns in *NAD5* (NADH dehydrogenase subunit 5) and *COB* (cytochrome b) in the mtDNA of JCM 15448 (S2 Fig).

**Table 2:** Genetic features of chromosomal DNA draft sequences and complete mitochondrial DNA in *C*. *auris* JCM 15448.

Type	Status	SeqID	CDS	rRNA	tRNA	Length (bp)	Average read depth^a^	Putative copy number^b^	Accession number
Chromosomal DNA	High quality draft sequence	contig_1	1,677	0	54	3,850,246	604	1	BGOX01000001
	High quality draft sequence	contig_2	1,385	0	42	3,155,023	626	1	BGOX01000002
	High quality draft sequence	contig_3	1,020	0	27	2,346,960	619	1	BGOX01000003
	High quality draft sequence	contig_4	389	3	4	943,590	629	1	BGOX01000004
	High quality draft sequence	contig_5	348	0	13	865,637	590	1	BGOX01000005
	High quality draft sequence	contig_6	387	0	11	861,415	617	1	BGOX01000006
	High quality draft sequence	contig_7	3	16	0	31,689	2,701	4	BGOX01000007
	High quality draft sequence	contig_8	10	0	0	27,068	676	1	BGOX01000008
	High quality draft sequence	contig_9	2	0	0	8,103	614	1	BGOX01000009
	High quality draft sequence	contig_10	1	0	1	6,957	723	1	BGOX01000010
	High quality draft sequence	contig_11	1	0	0	1,627	620	1	BGOX01000011
Mitochondrial DNA	complete sequence	mtDNA	15	2	32	27,071	5,561	9	AP018713

^a^The average read depth was calculated by illumina short-read mapping analysis using the bwa-mem program.

^b^The copy number was calculated by the number of each contig read depth divided by that of average read depth of total chromosomal DNA excluding contig_7.

### Comparative core genome and phylogenetic analysis of 133 *C*. *auris* strains

**Fig 1:**
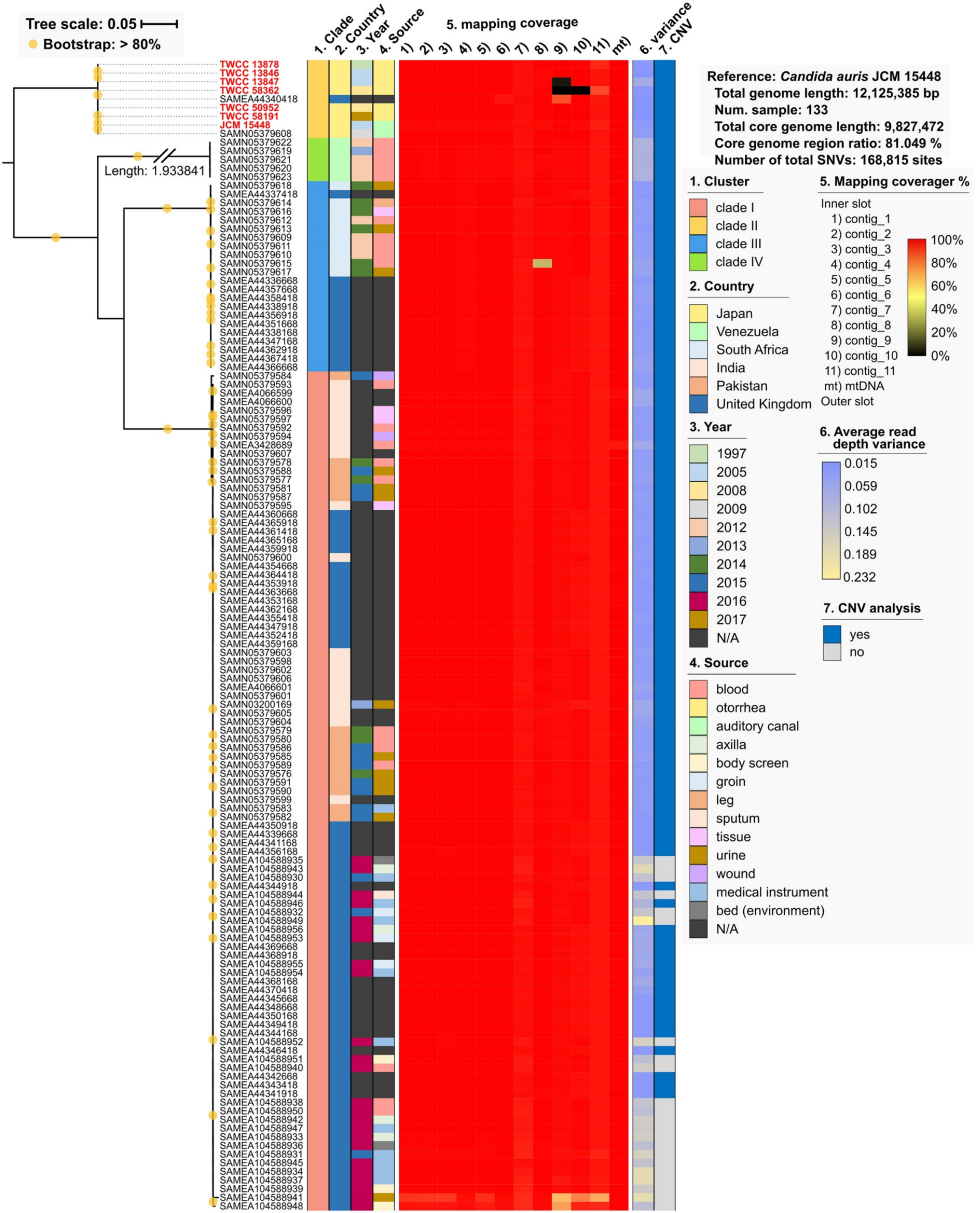
Comparative whole genome analysis of *C*. *auris* among seven Japanese strains and 126 strains deposited in SRA database. Phylogenetic analysis with SNVs on core genome regions. 168,815 SNVs were detected in 133 strains. The maximum-likelihood phylogenetic tree was constructed using RAxML version 8.2.0. The clade, metadata, and mapping analysis results are described in the color schemes on the right of the phylogenetic tree.

The SNVs on core genome regions were extracted using read-mapping analysis with the JCM 15448 genome as a reference. In this study, the core genome represents the genome sequences excluding repeat regions conserved in all strains by read mapping analysis. A total of 81.05% of the region was assigned as the core genome sequence among 133 *C*. *auris* strains, which included 7 Japanese strains that were characterized in the present study, and 126 available genome sequences in the SRA database (Fig 1 and S1 Dataset). The total number of SNVs on core genome regions was 168,815 among the 133 *C*. *auris* strains, and phylogenetic analysis using these SNVs indicated the existence of 4 distinct clades (Fig 1), which closely relate to the geographic location, and are consistent with a previous report [13,23,24]. The number of average pairwise SNVs between clade II and other clades ranged from 48,463 to 133,423. All Japanese strains, which were isolated from otorrhea or the auditory canal, and one strain (BioSample ID: SAMEA44340418) from the UK belonged to clade II (Fig 1), and the MLST allele profile of “a-a-a-a”, i.e., ST cluster 2 (Table 1 and S1 Dataset), was the same as that of *C*. *auris* strains isolated in Korea [21]. Phylogenetic analysis using mtDNA SNVs revealed the same phylogenetic relationship as that using the core genome sequence (S3 Fig), and mtDNA structures were highly conserved among strains in each clade (S4 Fig). Structural mtDNA variation was detected in *COX1* of strains in clade I, III, and IV, and these genes were divided into two regions by a putative intron. These results demonstrate that nucleotide substitution diversity is related to the geographic location in both chromosomal DNA and mtDNA.

The median pairwise SNV count for clade I, II, III, and IV was 43, 88, 27, and 7, respectively (S5 Fig). Mapping analysis indicated complete loss of contig_9 in two Japanese strains, TWCC 58362 and TWCC 13847, and loss of contig_10 in TWCC 58362 (Fig 1 and S2 Fig). The sequence of these contigs (9 and 10) could be acquired via horizontal gene transfer from other strains. These results suggest that the genetic variation within clade II is higher than that of other clades.

### Clade-specific accessory genes in *C*. *auris*

**Fig 2:**
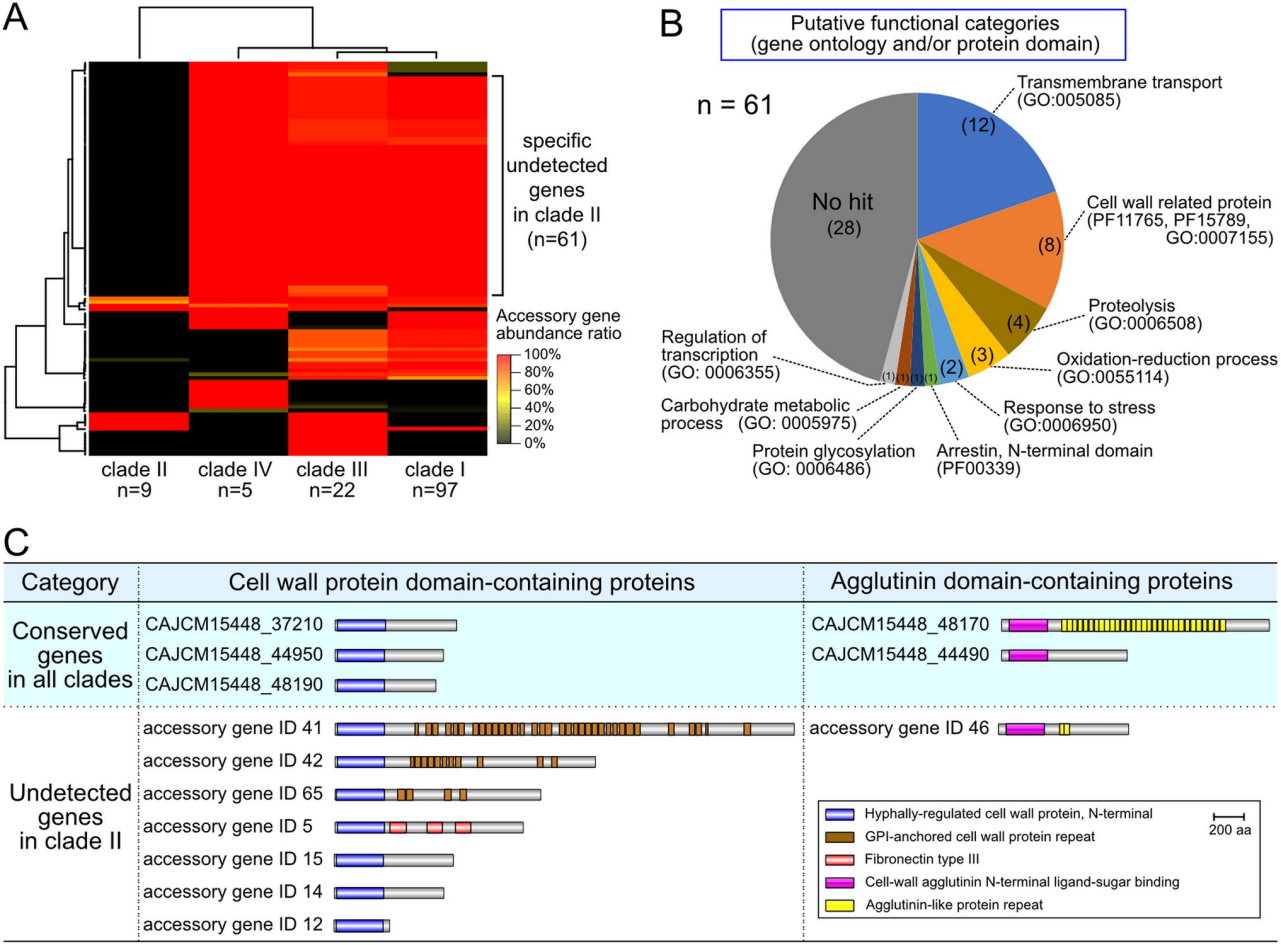
Pangenomic analysis of *C*. *auris* strains. (A) The abundance ratio of the accessory gene among the *C*. *auris* clades determined using hierarchical cluster analysis. The relative abundance is shown using color gradients. Sixty-one genes were determined to be specific unidentified genes in clade II. (B) Functional prediction analysis in specific unidentified genes of clade II. A pie chart showing the ratio of the 61 genes categorized by protein domain information and/or gene ontology terms. (C) List of specific unidentified genes, which are related to cell wall proteins, in clade II. The predicted functional domains of the amino acid sequences are described in schematic illustrations. In the pangenome of *C*. *auris*, ten and three proteins were predicted to be cell wall protein domain-containing proteins and agglutinin domain-containing proteins, respectively. Eight genes coding cell wall-related proteins remain unidentified in only clade II.

Mapping analysis indicated a partial gene loss in *C*. *auris*. To further investigate the genomic variation of *C*. *auris*, a pan-genomic approach was adopted, because read-mapping analysis as described above could bias the apparent gene composition based on the reference sequences. The unmapped reads from the read-mapping analysis were extracted and assembled, followed by BLASTN search with the nucleotide database and read depth checking. In each sample, excluding clade II strains, there was a strong correlation of the average read depth between mapping data of reference sequences and contigs that were related to other *C*. *auris* genomes assembled from unmapped reads (S1 Dataset). On the other hand, the read depth of contigs that seemed to originate from other organisms (i.e. bacteria, other fungi, etc.) are four times lower than the those of the *C*. *auris* genome (S1 Dataset). If these sequences are integrated in the *C*. *auris* genome by recombination, mating events, or HGT, these read depths must also be also the same as those of the *C*. *auris* genome (S6 Fig). The total length of contigs originating from other organisms is over than 5 Mb in three samples (SAMEA104588948, SAMN05379605, and SAMN05379606). However, these read depths are similar to or higher than those of *C*. *auris*. In this case, it is suggested that these sequences are generated from the contamination of original samples. Therefore, these results strongly suggested that 13 of the 133 deposited SRA samples could be contaminated with the genomic DNA of other organisms, including *C*. *glabrata* and *Cyberlindnera jadinii* (S1 Dataset). All nine strains in clade II had no unmapped reads, suggesting that there might be no additional gene acquisition by horizontal gene transfer (HGT) (S1 Dataset). The pan-genomic analysis revealed that the number of clusters against the total, core, and accessory genes was 5,336, 5,214, and 109, respectively. A heatmap analysis revealed that 61 of 109 accessory genes were not detected in clade II (Fig 2A and S2 Dataset). These 61 unidentified genes in only clade II were characterized using gene ontology and domains predicted from protein sequences as shown in Fig 2B. A total of 33 out of 61 genes were assigned to some functional category, which was predicted by InterProScan. Out of these, 12, 8, 3, and 2 genes belonged to transmembrane transporter, cell wall, oxidation-reduction, and stress response related proteins, respectively. The pan-genomic analysis showed that the cell wall proteins were classified into the two groups, i.e., hyphally regulated cell wall protein domain (HD) and agglutinin domain (AD)-containing proteins. Three HD and two AD- containing protein genes were conserved in all strains, while seven HD and one AD-containing protein genes were unidentified in clade II (Fig 2C). Intriguingly, these seven HD-containing proteins possessed unique domain structures (differences in the GPI-anchored cell wall protein repeat, and the existence of a fibronectin type III domain), suggesting that these predicted cell wall proteins might have different functions related to adhesion against various environmental surfaces.

### Clade-specific copy number variation among *C*. *auris* strains

**Fig 3:**
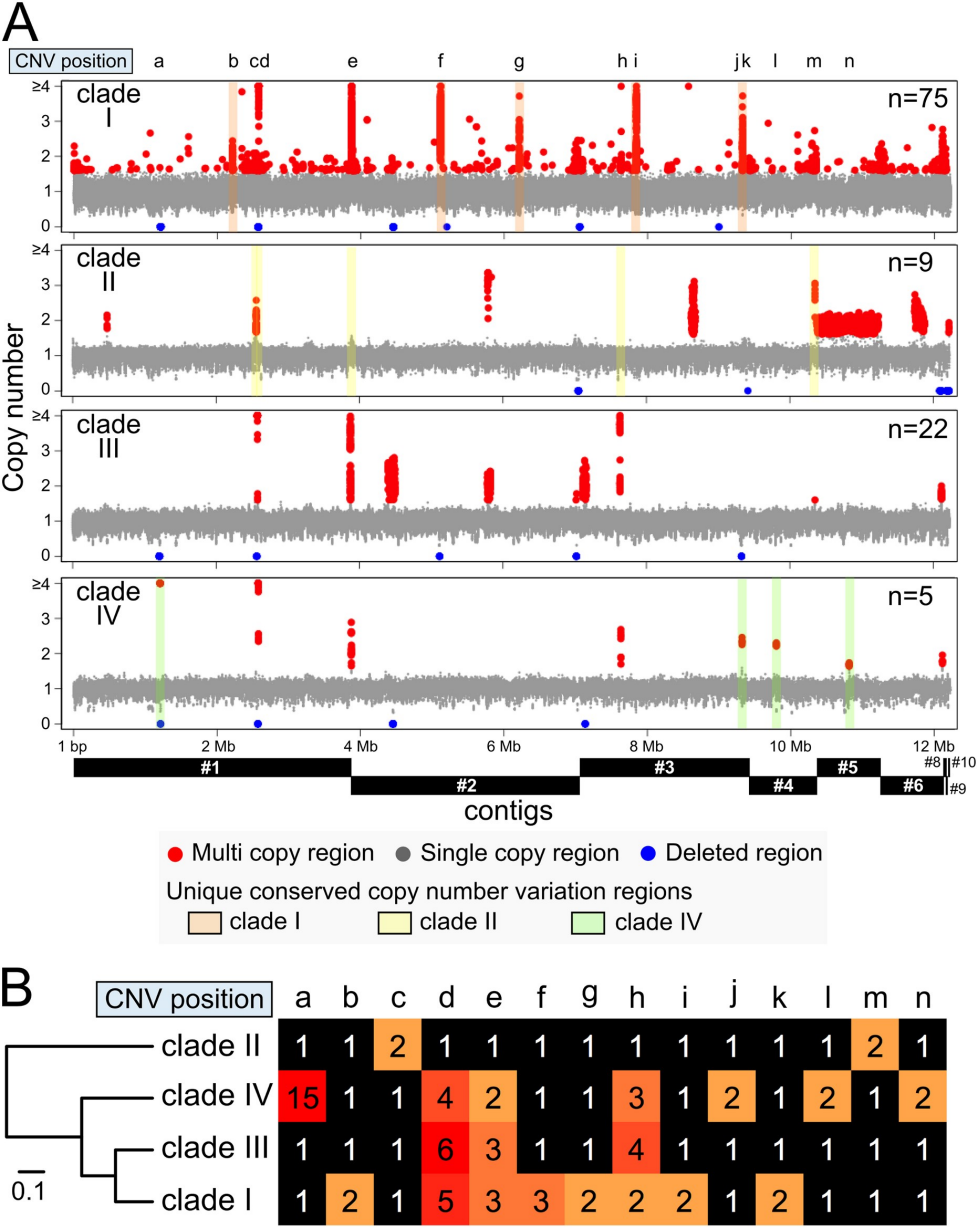
Comparison of copy number variations (CNVs) among four *C*. *auris* clades. (A) A graphic representing the CNV data excluding 22 high variance samples is merged in each clade. A high copy region was defined as having more than 1.6 copies. The conserved CNV regions in each clade are labeled with colored boxes as follows: orange: clade I, yellow: clade II, green: clade IV. Alphabets of small letter indicate the positions of conserved CNVs. CNVs greater than four copies are shown as four copies for ease of interpretation. (B) Phylogenetic tree analysis with conserved CNVs among four clades. The positions of CNV are displayed by alphabets as described above. The copy numbers are showed by a color gradient with black, orange and red. The detailed information of conserved CNVs are described in Table 3.

To characterize copy number variation (CNV), all samples were verified using the variance of read depth. The statistical analysis of variance of read depth indicated that 22 of 133 samples contained high variance, i.e., abnormal distribution of read mapping depth (Fig 1 and S7 Fig). These 22 samples belonged to the same BioProject, ID PRJE20230, and were excluded from further CNV analysis because of the potential existence of artificial biases in these DNA-Seq libraries. Out of the remaining 111 samples, two Japanese strains, TWCC 58362 and TWCC 13847, had a complete loss of contig_9, and TWCC 58362 also had a complete loss of contig_10 (Fig 1). However, the distribution of read mapping depth was stable in other contigs of these two samples. As contig_7 and mtDNA include multi-copy regions, these sequences were excluded from CNV analysis. An average copy number of more than 1.6 copies was denoted as a high copy region and was distinguished from single copy regions (S8 Fig). Comparative CNV analysis indicated the existence of unique conserved copy number regions in clade I, II, and IV (Fig 3A and S3 Dataset). In particular, two conserved high copy and three conserved single copy regions were observed in clade II vs. the other clades (Fig 3A and Table 3). The phylogenetic analysis with conserved CNVs also revealed that the number and frequency of CNVs in clade II were the most stable among the four clades (Fig 3B). Comparison of the phylogenetic tree using chromosomal SNVs, mitochondrial DNA SNVs, accessory gene patterns, and conserved CNV patterns indicated that the four trees had a similar topology pattern, and that clade II belongs to an out-group (S9 Fig). These results suggest that the genetic variation, which includes SNVs, CNVs and accessory gene patterns, is conserved within each clade, and that the genomic structures of clade II might be closely related to those of a common ancestor.

**Table 3:** Unique conserved copy number variations on chromosomal DNA among each *C*. *auris* cluster.

Description	Contig name	Region	Position (See Fig 3)	Average copy numbers^a^				Main CDS on CNV regions
				clade I	clade II	clade III	clade VI	
clade IV unique CNV	contig_1	1205001..1208000	a	1	1	1	15	CAJCM15448_05240 (hypothetical protein)
clade I unique CNV	contig_1	2211001..2212000	b	2	1	1	1	CAJCM15448_09730 (hypothetical protein)
clade II unique CNV	contig_1	2556001..2561000	c	1	2	1	1	CAJCM15448_11290 (hypothetical protein)
								CAJCM15448_11300 (putative ZIP zinc/iron transporter)
clade II unique CNV	contig_1	2573001..2578000	d	5	1	6	4	non coding region
clade II unique CNV	contig_2	1..4000	e	3	1	3	2	CAJCM15448_16780 (hypothetical protein)
clade I unique CNV	contig_2	1239001..1246000	f	3	1	1	1	CAJCM15448_22010 (putative group II intron reverse transcriptase/maturase)
clade I unique CNV	contig_2	2339001..2340000	g	2	1	1	1	CAJCM15448_26910 (hypothetical protein)
clade II unique CNV	contig_3	583001..584000	h	2	1	4	3	CAJCM15448_33140 (hypothetical protein)
clade I unique CNV	contig_3	794001..798000	i	2	1	1	1	CAJCM15448_34040 (putative group II intron reverse transcriptase/maturase)
clade IV unique CNV	contig_3	2273001..2274000	j	1	1	1	2	CAJCM15448_40520 (putative group II intron reverse transcriptase/maturase)
clade I unique CNV	contig_3	2277001..2279000	k	2	1	1	1	CAJCM15448_40520 (putative group II intron reverse transcriptase/maturase)
clade IV unique CNV	contig_4	384001..385000	l	1	1	1	2	non coding region
clade II unique CNV	contig_4	935001..936000	m	1	2	1	1	non coding region
clade IV unique CNV	contig_5	443001..445000	n	1	1	1	2	CAJCM15448_46580 (hypothetical protein)

^a^Number of copy numbers is indicated using rounding up digits after the decimal point to form an integer.

### Comparative genome analysis of clade II *C*. *auris*

**Fig 4:**
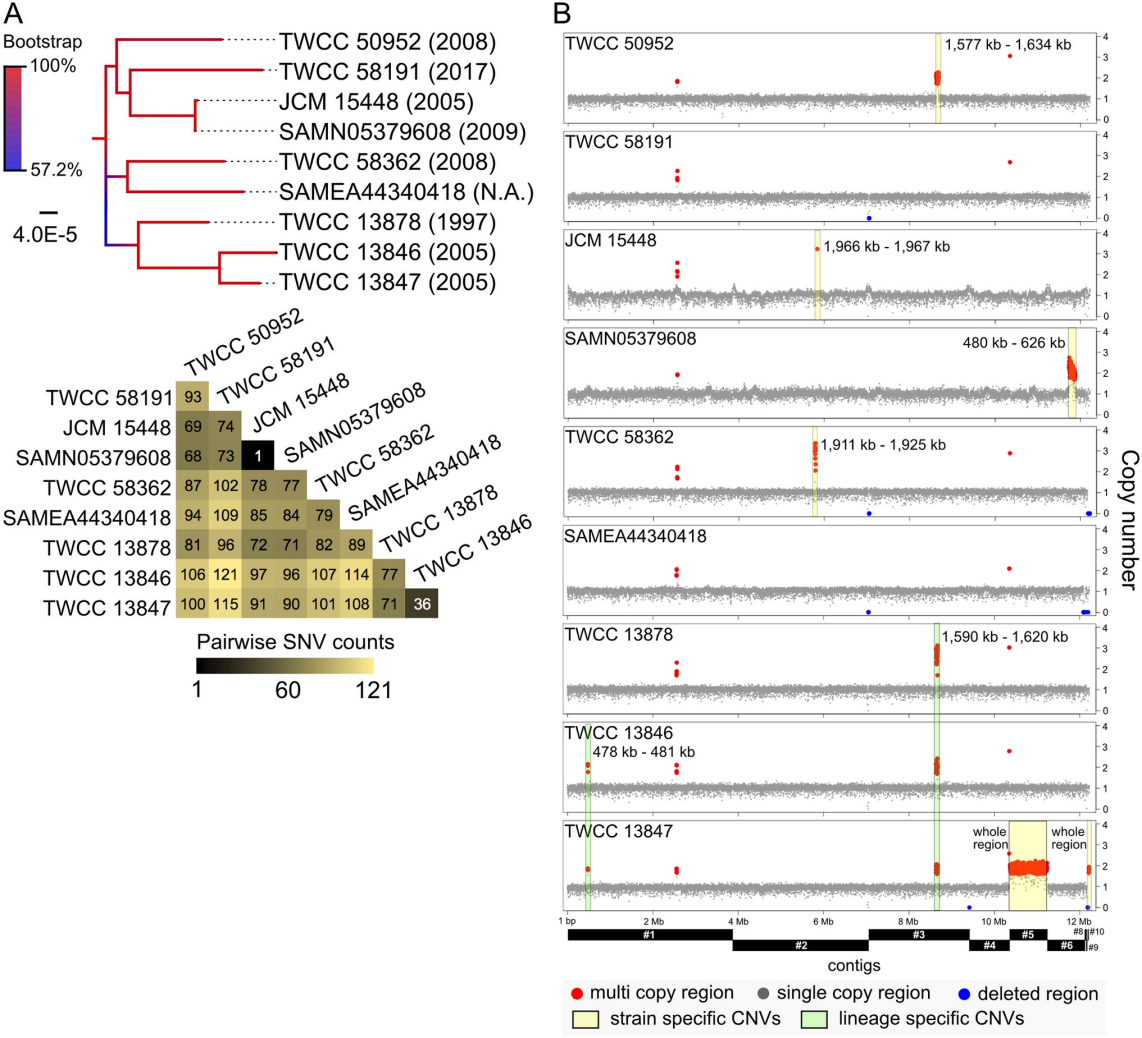
Comparative whole genome analysis of nine strains of clade II *C*. *auris*. (A) Core genome SNV phylogenetic tree of strains belonging to clade II. All-against-all comparisons of SNV counts are shown by a lower triangular matrix using a color gradient with black and yellow. (B) Comparative CNV analysis among strains in clade II. Unique CNV regions are labeled by yellow and light green boxes, and base range is noted on each contig.

More detailed comparative analysis was carried out between seven Japanese *C*. *auris* strains and two samples deposited in the SRA database. In this comparison, 313 SNVs were detected in clade II in total. Only one SNV was detected between JCM 15448 and B11220 (BioSample ID: SAMN05379608; geographic location: Japan) (Fig 4A), and this SNV was a nonsynonymous mutation (L244I) in putative O-succinyl benzoate-CoA ligase gene (CAJCM15448_24570). However, CNV differences were detected between these two strains (Fig 4B), suggesting that they may have originated from the same clonal lineage, but not the same strain. Our results and the results of previous reports indicate that the MICs of FLC in JCM 15448 and B11220 are 1 μg/ml and 4 μg/ml, respectively [14,25]. Although both strains were susceptible to FLC, the MIC of FLC in B11220 was four times higher than that of JCM 15448. The duplication of approximately 146 kb, comprising 63 genes, was detected in B11220, and two of these genes, CAJCM15448_50480 and CAJCM15448_50500, encode putative cytochrome P450 and putative phospholipid-translocating P-type ATPase (flippase), respectively. These data suggest that the genetic variations between JCM 15448 and B11220 might correlate to slight differences in susceptibility to FLC.

Mapping analysis revealed that there are several deleted regions and CNVs in clade II. These structural variations highlight strain- and lineage-specific features (Fig 4B). Notably, TWCC 13878, 13846, and 13847 belong to the same lineage and contain the CNV region from 1,590 to 1,620 kb on contig_3 (Fig 4B). Only two strains, TWCC 13846 and 13847, contain the CNV region from 478 to 481 kb on contig_1. However, TWCC 13847 possess other unique CNVs in contig_5, contig_9, and contig_10. These results indicate that some CNVs were conserved during the divergence of this lineage.

Isolates TWCC 13846 and 13847 were obtained from the left and right ear of a patient with chronic middle otitis on same date, respectively. However, 36 sites of SNVs and three regions of CNVs were detected between these two strains, suggesting that the common ancestral clone might have acquired different mutations with a slight variation in the environment over a long time or a population of different genotypes might have colonized in each ear.

In the present study, only one isolate (TWCC 58362) was resistant to FLC (Table 1). It was previously reported that mutation or upregulation of *ERG* (*ERG3* and *ERG11*) were associated with azole resistance in *C*. *albicans* and *C*. *auris* [13,24,26,27]. All ergosterol biosynthesis-related genes, including *ERG3* and *ERG11*, are present in the JCM 15448 strain (S2 Table). Somewhat paradoxically, nucleotide mutations were not detected in these ergosterol biosynthesis genes and the adjacent regions in the TWCC 58362 strain. Comparative analysis of clade II strains identified 86 unique mutation sites in TWCC 58362, which are therefore novel resistance genetic markers for FLC (S4 Dataset). In particular, nonsynonymous mutations in 20 genes, multi copy regions in 10 genes, and gene loss of two genes was identified in TWCC 58362. The multi copy genes were included in one region of approximately 14 kb in contig_2, and the number of duplications in this CNV were approximately three times that of single copy regions (Fig 4B and S3 Dataset). These results suggest that specific mutation sites, including CNVs in TWCC 58362, might relate to an uncharacterized antifungal resistance mechanism.

## Discussion

In the present study, we obtained high-quality draft genome sequences, including complete mtDNA sequences, for *C*. *auris* isolated in Japan. Comparative analysis revealed that the synteny and structure of mtDNA dramatically changed among *Candida* and related species and that *C*. *auris* mtDNA might contain introns in *NAD5* and *COB* genes (S2 Fig). Mitochondrial DNA structures have great evolutionary variability, even in closely related fungal species [28], and therefore, the mtDNA of *C*. *auris* may have also evolved in a unique manner in the genus *Candida*. The mtDNA sequences except for the *COX1* region are highly conserved in *C*. *auris* strains, and the phylogenetic relationship of mtDNA is similar to that of the chromosomal DNA (Fig 1 and S3 Fig). This suggests that the nuclear and mitochondrial DNA of *C*. *auris* might have followed parallel evolution, albeit at different evolutionary rates.

Although the monophyletic clades of *C*. *auris* have disseminated in specific biogeographic locations, including Japan (Fig 1), the median of pairwise SNV counts of clade II is significantly higher than that of other clades (S5 Fig). All Japanese strains used in this study were isolated from patients with non-invasive, sporadic cases of otitis media, suggesting that the sporadic nature of these cases might be explained by SNV genomic analysis. The CDC issued a warning related to the global emergence of invasive infections caused by multidrug-resistant *C*. *auris* in 2016 (https://www.cdc.gov/fungal/candida-auris/candida-auris-alert.html), and cases of large-scale nosocomial invasion have been reported in more than 30 countries on six continents [22,29,30]. On the other hand, all Japanese *C*. *auris* strains were isolated from ear specimen and not from invasive mycoses [22], highlighting the differences in virulence and/or environmental viability might exist between *C*. *auris* clinical isolates from Japan and other countries. However, the comparative genomic analysis revealed several unidentified genes in the Japanese strains (Fig 2A), including transmembrane transport proteins, oxidation-reduction related proteins, and stress response proteins, which might be important for coping with environmental stress (Fig 2B). Pangenomic analysis also suggested that the features of clinical isolates that caused outbreaks or invasive infections might be related to the number of and/or variations to genes encoding cell wall proteins, which were classified into two groups: 1) hyphally-regulated cell wall protein domain (HD) and 2) agglutinin domain (AD)-containing proteins. It was previously reported that HD-containing protein in *C*. *albicans*, named Hyr1, is an important virulence factor mediating resistance to neutrophil killing, and is required for pathogenesis during *Candida* mucosal biofilm infections [31–33]. HD-containing proteins are comprised of various domain structures and are different lengths, suggesting that these cell wall proteins could serve different functions, including mediating virulence. Eight agglutinin-like sequence (Als) family proteins are made up of an agglutinin domain and agglutinin-like protein repeats [34,35], and one of these eight proteins, Als3, is linked to epithelial cell interaction [36]. Thus, we speculate that the AD-containing proteins of *C*. *auris* might be also related to cell adhesion. All *C*. *auris* strains from Japan, which lack multiple cell wall proteins, were detected from ear specimens but not invasive mycoses, leading us to speculate that these eight cell wall proteins are not essential proteins and might act as virulence factors, including cell adhesion ability.

Clinical information and our comparative genomic data suggest that the Japanese strains isolated from patients with otitis media might be closely related to the most common ancestral type rather than other strains isolated elsewhere. HGT, gene duplications, gain and loss are an important evolutionary process in prokaryotes [37]. It was suggested that HGT is also important evolutional mechanisms in unicellular eukaryote, particularly in fungi [38]. Some strains that caused outbreaks or invasive infections might therefore have evolved through the acquisition of function with HGT against environmental stress and host cell interactions. To demonstrate this hypothesis, further genomic comparisons between invasive and non-invasive isolates belonging to clade II may identify the genetic factors underlying invasive and multidrug-resistant phenotypes.

Previous reports have described the relationship between SNV patterns and geographic locations [13,23,24]. In the present study, we revealed that the geographic location of *C*. *auris* strains closely related to genetic features, including nucleotide substitutions, accessory gene patterns, and CNVs (Figs 1, 2A and 3B). CNV patterns were also conserved in a small lineage of clade II (Fig 4B). We therefore speculate that nucleotide substitutions, and gene acquisition and duplication events might also be related to the evolutionary steps associated with geographic segregation of *C*. *auris*. In addition, our data suggests that clade II might be an ancestral type because of the low frequency of CNVs observed in clade II compared to other clades.

In the present study, one of the seven Japanese strains, TWCC 58362, was resistant to FLC (Table 1). This strain had no mutations detected in *ERG*, which have previously been associated with azole resistance [13,24,26,27]. Although biofilm formation is associated with FLC resistance [14], it has been reported that *C*. *auris* isolates from clade II from ear specimens did not form biofilms [39]. Comparative genomic analysis revealed that TWCC 58362 has unique mutation sites, which included SNVs, indels, and CNVs (S4 Dataset), suggesting that other mechanisms for drug resistance might exist in this strain. In *C*. *albicans*, chromosome 5 monosomy, which is related to CNVs, confers reduced susceptibility to several antifungals and shifts the ratio such that a diminished dose of genes for negative regulation determines tolerance to caspofungin [40,41]. The same phenomenon might not be applicable to *C*. *auris*, because *C*. *auris* is haploid while *C*. *albicans* is diploid. Comparative analysis between JCM 15448 and B11220 revealed that duplication of putative cytochrome P450 (CAJCM15448_50480) and putative phospholipid-translocating P-type ATPase (CAJCM15448_50500) in B11220 might be related to the degree of susceptibility to FLC. Ergosterol is one of the important components of the fungal cell membrane, and FLC bind to Erg11, which is a member of the cytochrome P450 family, to inhibit ergosterol biosynthesis [42–44]. A family of P-type ATPases has previously been associated with transport or flipping of phospholipids across cell membranes [45]. Therefore, duplications of these genes (CAJCM15448_50480 and CAJCM15448_50500) through CNVs might be related to the degree of susceptibility to FLC via homeostasis of ergosterol biosynthesis and/or stability of the cell membrane structure. Our data hints that the variability of CNVs in *C*. *auris* might represent a new mechanism of antifungal drug resistance.

FLC resistance in *C*. *auris* has been reported in several countries, and our data represent the first case of FLC resistance in a clinical strain in Japan. Azole antifungals are used for both human and animal pharmacotherapy and as an agricultural pesticide, antifouling coating, and timber preservative [46]. Spatiotemporal data from the United States Geological Survey (USGS) shows that the consumption of agricultural triazole is increasing in the USA (https://water.usgs.gov/nawqa/pnsp/usage/maps/compound_listing.php). Consumption of triazoles and diazoles, which are used as pesticides, is reported by the Food and Agriculture Organization of the United Nations (FAO), has increased in Japan, Germany, France, the UK, Poland, Spain, and Italy, every year (FAOSTAT: http://www.fao.org/faostat/en/?#home). Although the trend of azole usage in Japan is similar to those of Spain and Italy, the total consumption of azoles in Japan is 2 to 8.7 times lower than those of Germany, France, the UK, and Poland. The number of reports of azole-resistant fungal species has increased in many counties [46], although the direct relationship between increased antifungal drug resistance in fungi and the consumption of antifungal drugs is not currently known. The percentage of isolates that are not susceptible to FLC, or with decreased susceptibility to FLC, is positively correlated with the usage of FLC at individual hospitals in Korea [47]. We speculate that the frequency of usage of antifungal drugs in humans and environments might be related to the appearance of FLC resistance in *C*. *auris* in a biogeographic location-dependent manner.

In conclusion, we present a high-quality draft chromosomal and complete mitochondrial DNA sequences of *C*. *auris* JCM 15448 strain, and performed a large-scale comparative genomic analysis among *C*. *auris* strains isolated from several countries across three continents. Our data suggest that *C*. *auris* with stable genomic structures, which conserved gene acquisition and copy number variations, have been expanding from Asia to each biogeographic location, followed by an increasing epidemic in the world, with the exception of Japan. We speculate that Japanese isolates belonging to clade II might have evolved to adapt to the host, and have a propensity for the ear, resulting in a non-invasive clinical phenotype, which is supported by our genomic and clinical data. Our comparative analysis among Japanese isolates revealed that antifungal drug resistance in *C*. *auris* might correlate to nonsynonymous substitution by point mutation and pseudogenization by indels, as well as gene duplication by CNVs. Finally, we demonstrated that the specific genetic background of *C*. *auris* in East Asia, including Japan, is obviously distinct from those of other countries, and that FLC resistant strains belonging to the East Asian clade, clade II, possess marked genetic variations related to resistance mechanisms against antifungal drugs.

## Materials and methods

### Fungal growth conditions and identification

Two strains of *C*. *auris*, JCM 15448 and TWCC 58191, were previously isolated in Japanese hospitals [18,19]. Ascomycetous yeast strains, isolated from otorrhea of patients with chronic otitis media in Japan from 1997 to 2017 (Table 1), were cultured using Sabouraud dextrose agar (Becton Dickinson, Baltimore, MD, USA) for 2 days at 35°C. For identification of these clinical isolates, DNA extraction was performed using Isoplant II DNA extraction kit (Nippon Gene inc., Tokyo, Japan), direct-sequencing of the 26S D1/D2 domain and ITS region, and a BLASTN homology search against known sequences.

### Antifungal susceptibility test

The antifungal susceptibility test was performed according to the Clinical and Laboratory Standards Institute (CLSI) broth microdilution methods M27-A3 [48]. We used tentative breakpoints for *C*. *auris* proposed by the CDC (https://www.cdc.gov/fungal/candida-auris/c-auris-antifungal.html) and Lockhart *et al*. [13]. Briefly, interpretive breakpoints for *C*. *auris* were defined based on the breakpoints established for other closely related *Candida* species as follows: micafungin (MCFG) at ≥ 4 μg/mL, caspofungin (CPFG) at ≥ 2 μg/mL, amphotericin B (AMB) at ≥ 2 μg/mL, 5-fluorocytosine (5FC) at ≥ 128 μg/mL, fluconazole (FLC) at ≥ 32 μg/mL, and voriconazole (VRC) at ≥ 2 μg/mL.

### Whole genome sequencing

To prepare long-chain genomic DNA isolated from JCM 15448, DNA extraction was performed as described previously [49]. Briefly, fungal cells were cultured in 10 ml RPMI1640 medium for 2 days, suspended in 450 μl Tris-EDTA (TE) buffer. The cell suspension was supplemented with 50 μl SDS and 500 μl phenol/chloroform, followed by bead-beating for 10 min by vortexing. After centrifugation at 17,400 x g for 5 min, the upper aqueous phase was subjected to electrophoresis on a 1% TAE agarose gel, followed by purification of long size DNA (15-40kb) using a Zymoclean large-fragment DNA recovery kit (Zymoresearch, Irvine, CA, USA). Short read and long read DNA libraries with purified DNA were constructed using QIAseq FX DNA Library Kit (QIAGEN, Hilden, Germany) and the SMRTbell template prep kit 1.0 (PacBio, Menlo Park, CA, USA) according to the manufacturer instructions, followed by whole-genome sequencing using NextSeq (Illumina, San Diego, CA, USA), MiSeq (Illumina), and Sequel (PacBio).

### *De novo* assembly and annotation

Polishing of pre-assembled long reads was performed using Falcon version 0.3.0, followed by *de novo* assembly with polished long reads using Canu version 1.4 [50], Minimap version 0.2-r124 [51], Racon version 1.1.0 [52] and Circulator version 1.5.3 [53]. Error correction of the tentative complete or draft sequence was performed using Pilon version 1.18 [54] with Illumina short reads excluding adapter and low-quality sequences, which were trimmed using Fastq-mcf version 1.04.636 [55] and Sickle version 1.33 (https://github.com/najoshi/sickle). *De novo* assembly with short reads was performed using A5-miseq version 20140604 [56]. Gene prediction of complete or high-quality draft sequences was performed as follows: GeneMark-ES version 4.21 [57] using the fungal genome mode parameter against protein coding sequence (CDS); Aragorn version 1.2.38 [58] with default against tRNA; BLASTN search with rRNA sequences of *C*. *albicans* SC5314 (assembly accession number: GCA_000182965.3) against rRNA. Annotation of CDS was performed using Hmmscan in the HMMer software package version 3.1b2 [59] with TIGRFAMs database [60], RPS-BLAST [61] with the COG database [62] and InterProScan v49.0 [63]. The multi locus sequence typing (MLST) analysis was performed using a BLASTN similarity search using 4 allele sequences and allele profiles, which were reported by Kwon *et al* [21].

### Single nucleotide variation (SNV) detection and core-genome phylogenetic analysis

Publicly available short read sequencing data for *C*. *auris* was retrieved from the SRA database hosted by the NCBI in August 2018. A total of 133 samples (7 from the present study and 126 from the public database) were used in SNV detection analysis. Publicly available data of strains isolated from six countries (India, Japan, Pakistan, South Africa, the UK, and Venezuela) were included in seven projects {BioProject ID: PRJEB14717 (not published), PRJEB20230 [24], PRJEB21518 (not published), PRJEB9463 [64], PRJNA267757 [65] and PRJNA328792 [13,25]}. Read mapping was performed using bwa-MEM [66] with default parameters against the JCM 15448 genome sequence as a reference, followed by variant calling using VarScan v2.3.4 [67]. The repeat sequences in reference sequences were detected using NUCmer [68] with default parameters. The "core genome" was defined as the conserved region of the JCM 15448 genome among all strains and excluding repeat regions. Variant sites on repeat regions and low read depth regions, i.e., deleted regions, were excluded from the final SNV dataset. The concatenated sequences constructed from SNVs were used to construct a maximum-likelihood phylogenetic tree using RAxML version 8.2.0 with GTRCAT model and 1,000 bootstrap replicates [69]. A pairwise SNV distance matrix was created using Snp-dists (https://github.com/tseemann/snp-dists). The phylogenetic tree was visualized using Interactive Tree Of Life (iTOL) version 3 [70].

### Copy number variation (CNV) analysis

The mapping method with Illumina short reads was performed as described in the above subsection. The average coverage read depth with a window size of 1,000 bp (non-overlapping windows) was analyzed using igvtools version 2.3.98 [71] with the following parameters: z7 and w1000. The copy number ratio in 1,000 bp was calculated with the following formula: copy number ratio in 1,000 bp = read depth in 1,000 bp/average read depth of chromosomal contigs, excluding contig_7 which had tandem repeats of rRNA gene clusters. The scatterplot of CNV data was visualized using the plot function of R program.

### Accessory gene analysis

Unmapped reads in each sample were collected from mapping data followed by *de novo* assembly using A5-miseq. These contigs were searched using Megablast with the nucleotide database, and the taxonomic classification of contigs was validated using MEGAN version 6 [72], to eliminate putative contamination contig data. Gene prediction of the validated contigs was performed using GeneMark-ES as previously described. To generate orthologous gene clusters among genes predicted from unmapped contigs and JCM 15448 contigs, clustering analysis based on amino acid sequences was performed using USEARCH version 8.1.1812 [73] with the following parameters after sorting by sequence length: id = 0.9, minqt = 0.5, maxqt = 2, minsl = 0.5, query_cov = 0.5, and target_cov = 0.5. The database for each sample was constructed with consensus sequences, which were converted from mapping data, and from contigs, which were assembled from unmapped reads. The nucleotide sequences of these orthologous gene clusters were searched using Megablast against database of each sample. For hierarchical clustering analysis, the abundance ratio of accessory genes was calculated for each *C*. *auris* clade, and clustered using the amap package in R with the distance matrix computation using a Pearson distribution and hierarchical clustering using Ward’s method. The heatmap with dendrograms was drawn using heatmap.2 in the R package gplots. In this pan-genomic analysis, we defined a conserved gene as shared in ≥ 129 strains, and an accessory gene as shared in ≥ 4 strains, because all sequences are draft genomes and several contain DNA contamination.

### Data deposition

The sequences of JCM 15448 are available in DDBJ/EMBL/GenBank with the following accession numbers: chromosomal DNA of the draft genome, BGOX01000001-BGOX01000011; complete mtDNA, AP018713. Raw read sequence data have been deposited in DRA database under the following accession numbers: JCM 15448, DRR129819 and DRR129826; TWCC 13846, DRR129820; TWCC 13847, DRR129821; TWCC 13878, DRR129822; TWCC 50952, DRR129823; TWCC 58191, DRR129824; and TWCC 58362, DRR129825 (as shown in Table 1).

## Supporting information

S1 Dataset Summary of metadata and genomic analysis results for samples deposited in SRA database. (XLSX)

S2 Dataset List of accessory genes among 133 *C*. *auris* strains using pangenomic and hierarchical clade analysis. (XLSX)

S3 Dataset List of detected copy number variation regions among 111 *C*. *auris* strains. (XLSX)

S4 Dataset Unique variation sites of TWCC 58362 in comparison among clade II. (XLSX)

S1 Fig Experimental procedures for draft chromosome and complete mitochondrial sequencing of *C*. *auris* JCM 15448. The summary of long- and short-read information is described in the left panel. The histogram indicates read length distribution of subreads and polished long reads. The summary of *de novo* assembly information is described in the right panel.
(TIFF)

S2 Fig Comparative analysis of mitochondrial DNA among *C*. *auris* and related species. Schematic representation and comparative analysis of complete or draft mitochondrial DNA sequences among *Candida albicans* SC5314, *Clavispora lusitaniae* CBS 6936, *C*. *auris* JCM 15448^T^, *C*. *duobushaemulonii* B09383 and *C*. *haemulonii* B11899. Pairwise alignment was performed using TBLASTX, followed by visualization using Easyfig version 2.1 with following parameters: minimum length of blast hits, 30; maximum e-value, 1e-10; minimum identity value, 60. The red bars between mtDNA sequences represent individual amino acid sequences matches translated from nucleotide sequences. Light blue fragmented arrows represent the coding sequences (CDS) including intron predicted by comparative analysis.
(TIFF)

S3 Fig Phylogenetic analysis of mitochondrial DNA among 133 *Candida auris* strains. Twenty-eight SNVs were detected in 133 strains. The maximum-likelihood phylogenetic tree was constructed using FastTree version 2.1.10. Information of cluster type, metadata is described in color schemes on outside slots of the phylogenetic tree. The constructed clusters are closely similar to SNV trees with whole genome.
(TIFF)

S4 Fig Schematic representation and comparative analysis of complete or draft mitochondrial DNA sequences of four *C*. *auris* clusters and *C*. *haemulonii* B11899. Pairwise alignment was performed using BLASTN, followed by visualization using Easyfig version 2.1 with parameters described in S1 Fig. The mtDNA structures was conserved in each cluster.
(TIFF)

S5 Fig Boxplot of pairwise SNV counts among 4 clads. All-against-all comparisons of SNV counts were performed among each clade. Statistical analysis was performed using Wilcoxon rank sum test; the difference was statistically significant (*p* < 0.001) among all clusters. The boxplot indicates that median of pairwise SNV of clade I, III and IV is lower than those of clade II because of including outbreak case.
(TIFF)

S6 Fig Examples of scatter plots between average read depth and contig length. The contigs assembled with unmapped reads against reference sequences were analyzed by BLASTN, followed by taxonomic classification of contigs. These data reveal that the average read depth of non-*C*. *auris* contigs show different distributions from those of *C*. *auris* contigs.
(TIFF)

S7 Fig Statistical analysis of read mapping variance data for CNV analysis. This graph shows a histogram of read mapping variances. Twenty-two samples were rejected in CNV analysis because of abnormal distribution of read mapping depth. These all samples are included in same BioProject ID (PRJEB20230).
(TIFF)

S8 Fig Statistical analysis of short read mapping data for CNV analysis. This graph shows a histogram of average copy number in 111 samples which are collected by mapping variance analysis. Error bars represent standard deviation. The statistical data shows that lower than approximately 1.5 and more than 1.6 copy numbers are single and high copy, respectively.
(TIFF)

S9 Fig Phylogenetic tree analysis with chromosomal SNVs, mitochondrial DNA SNVs, accessory gene patterns and conserved CNVs among four clades. These trees indicate similar topology pattern, and the clade II belongs out of group against clade I, III and IV.
(TIFF)

S1 Table Summary of genome assembly statistics of *C*. *auris* strains. (PDF)

S2 Table Summary of antifungal drug resistance related gene of *C*. *albicans* and protein homology search with *C*. *auris* JCM 15448. (PDF)
