# Supplementary figures and images for: Clade II Candida auris possess genomic structural variations related to an ancestral strain
Source: PLoS One. 2019 Oct 9;14(10):e0223433. doi: 10.1371/journal.pone.0223433 (PMC6785063; doi:10.1371/journal.pone.0223433)

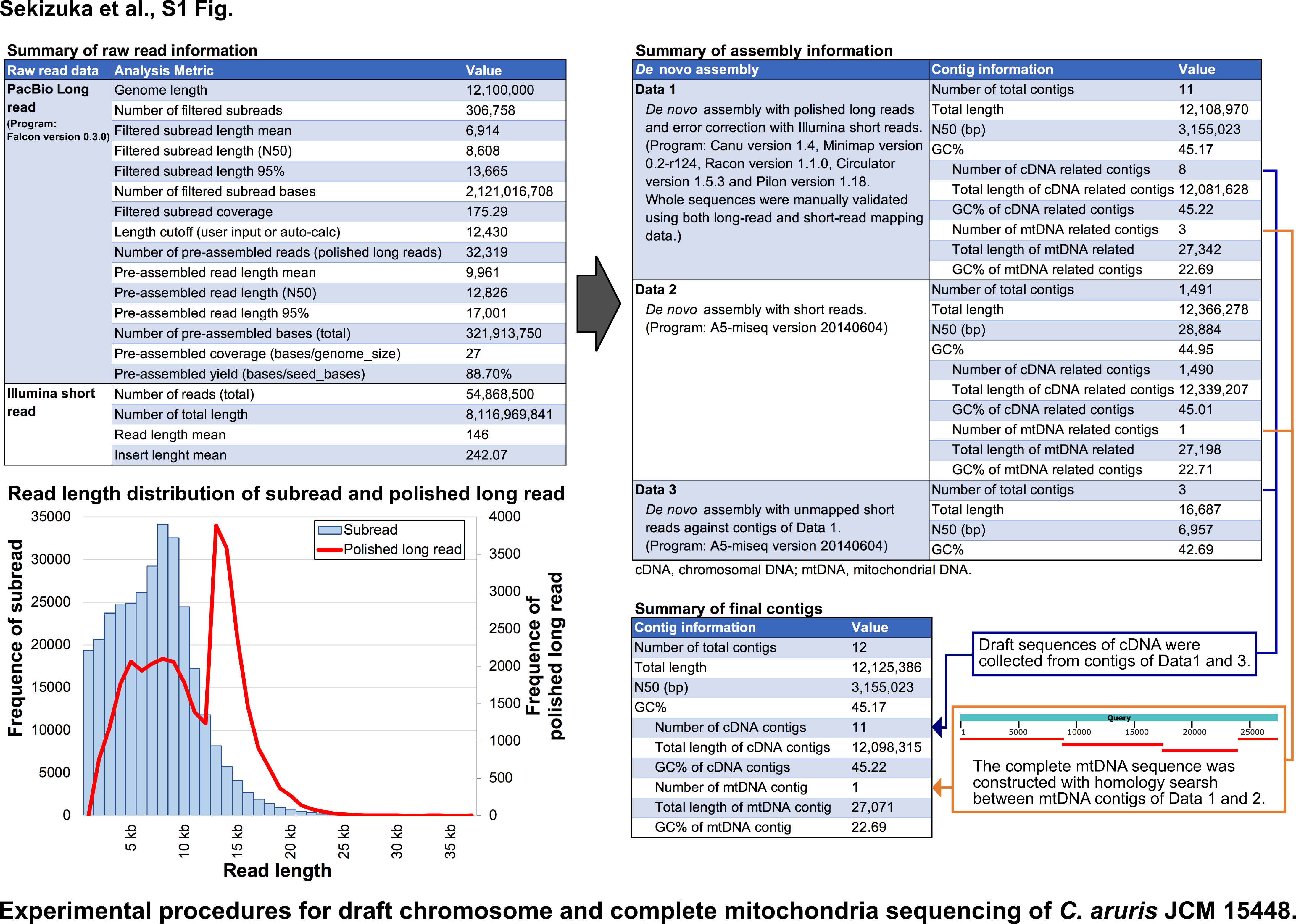

Supplement: S1 Fig — The summary of long- and short-read information is described in the left panel. The histogram indicates read length distribution of subreads and polished long reads. The summary of de novo assembly information is described in the right panel. (TIFF) [file pone.0223433.s005.tiff]

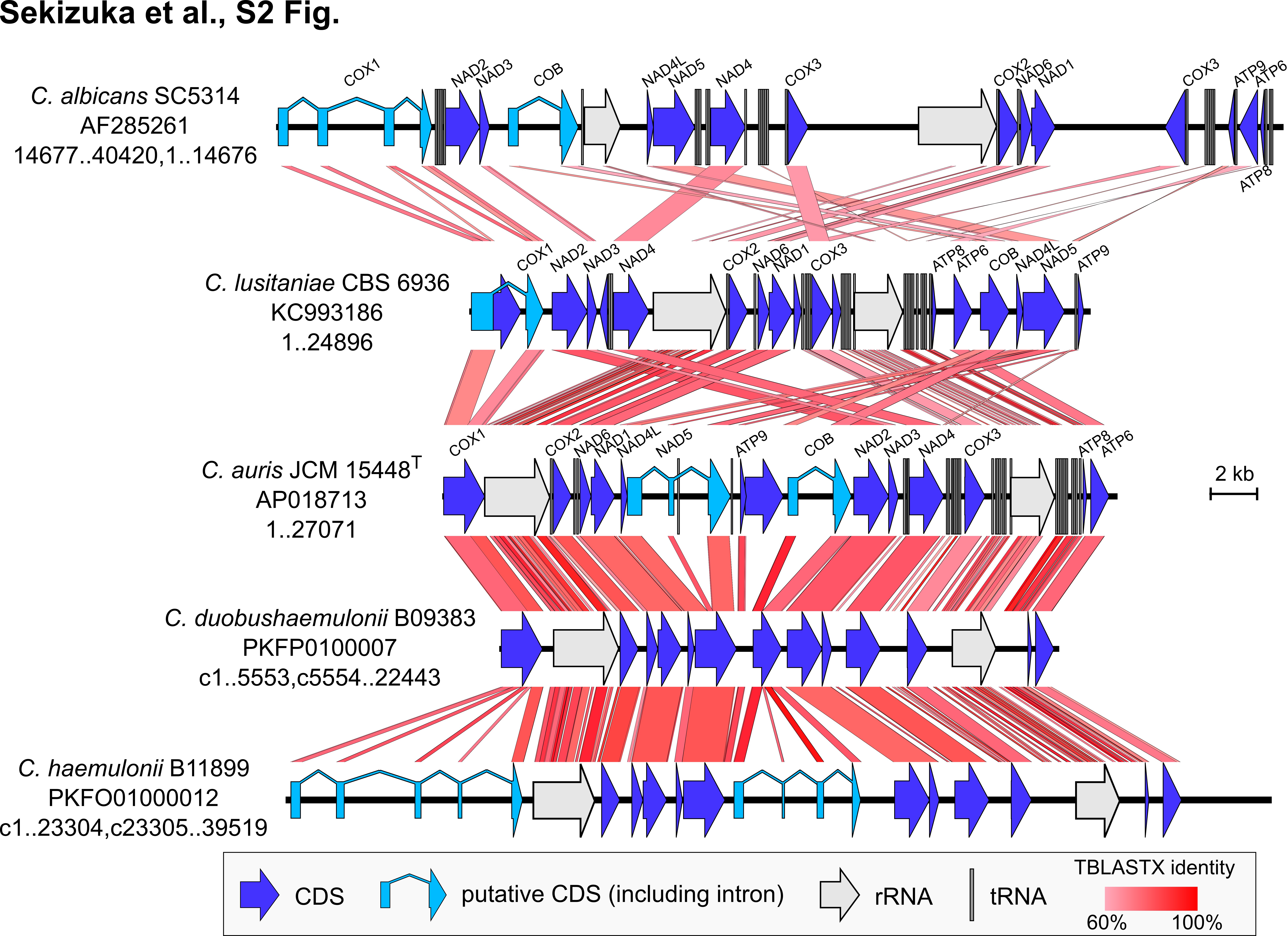

Supplement: S2 Fig — Schematic representation and comparative analysis of complete or draft mitochondrial DNA sequences among Candida albicans SC5314, Clavispora lusitaniae CBS 6936, C. auris JCM 15448T, C. duobushaemulonii B09383 and C. haemulonii B11899. Pairwise alignment was performed using TBLASTX, followed by visualization using Easyfig version 2.1 with following parameters: minimum length of blast hits, 30; maximum e-value, 1e-10; minimum identity value, 60. The red bars between mtDNA sequences represent individual amino acid sequences matches translated from nucleotide sequences. Light blue fragmented arrows represent the coding sequences (CDS) including intron predicted by comparative analysis. (TIFF) [file pone.0223433.s006.tiff]

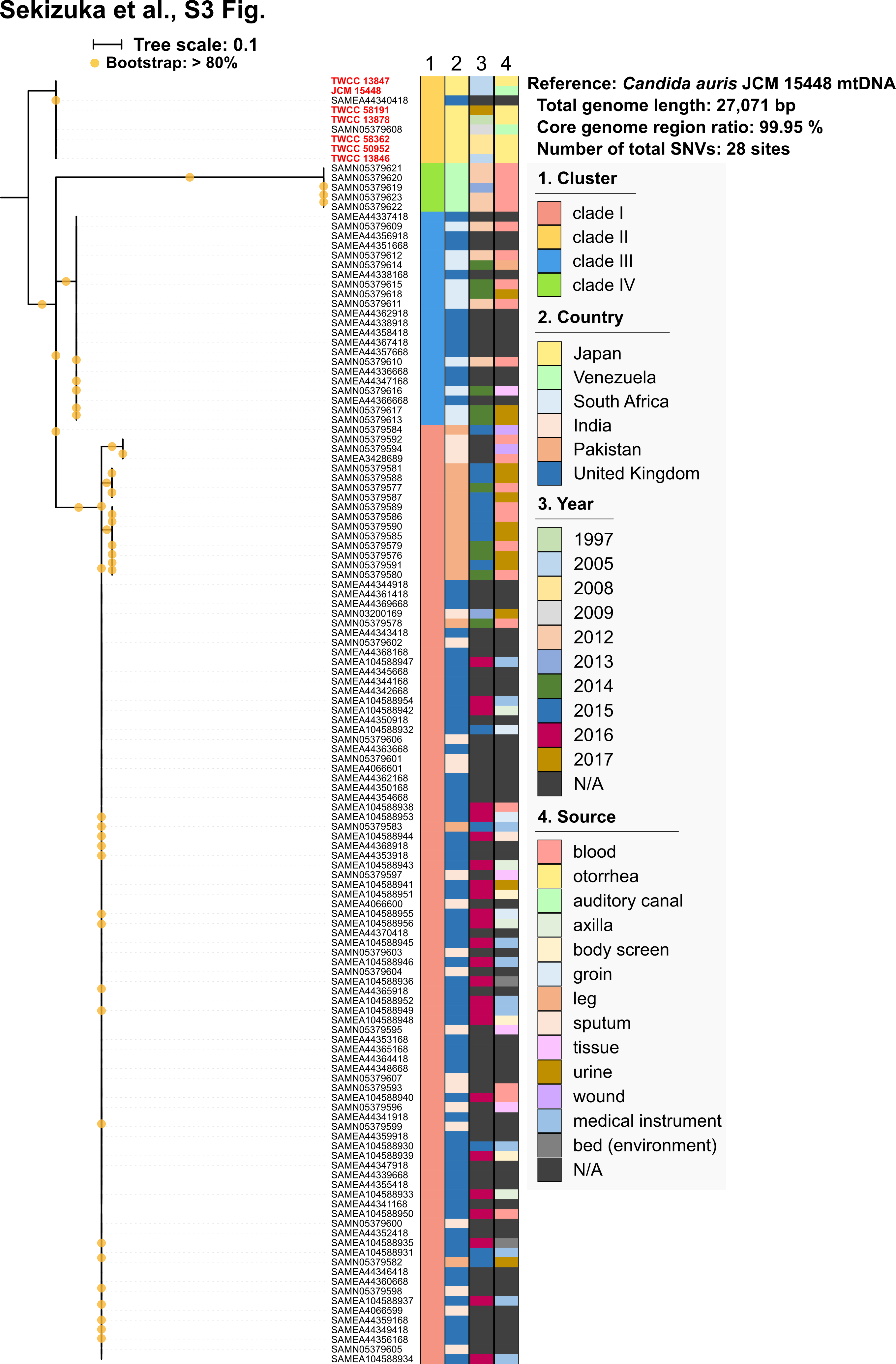

Supplement: S3 Fig — Twenty-eight SNVs were detected in 133 strains. The maximum-likelihood phylogenetic tree was constructed using FastTree version 2.1.10. Information of cluster type, metadata is described in color schemes on outside slots of the phylogenetic tree. The constructed clusters are closely similar to SNV trees with whole genome. (TIFF) [file pone.0223433.s007.tiff]

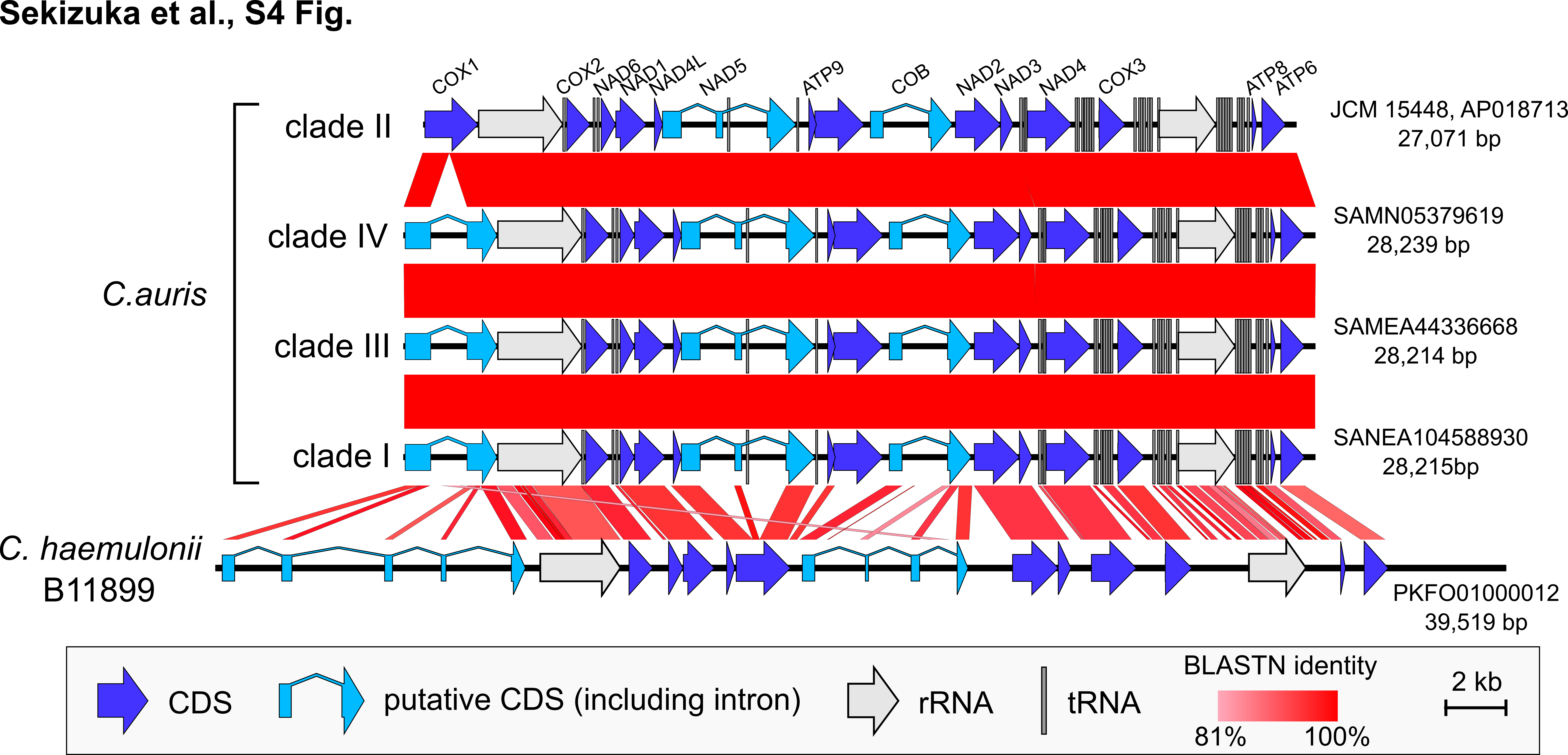

Supplement: S4 Fig — Pairwise alignment was performed using BLASTN, followed by visualization using Easyfig version 2.1 with parameters described in S1 Fig. The mtDNA structures was conserved in each cluster. (TIFF) [file pone.0223433.s008.tiff]

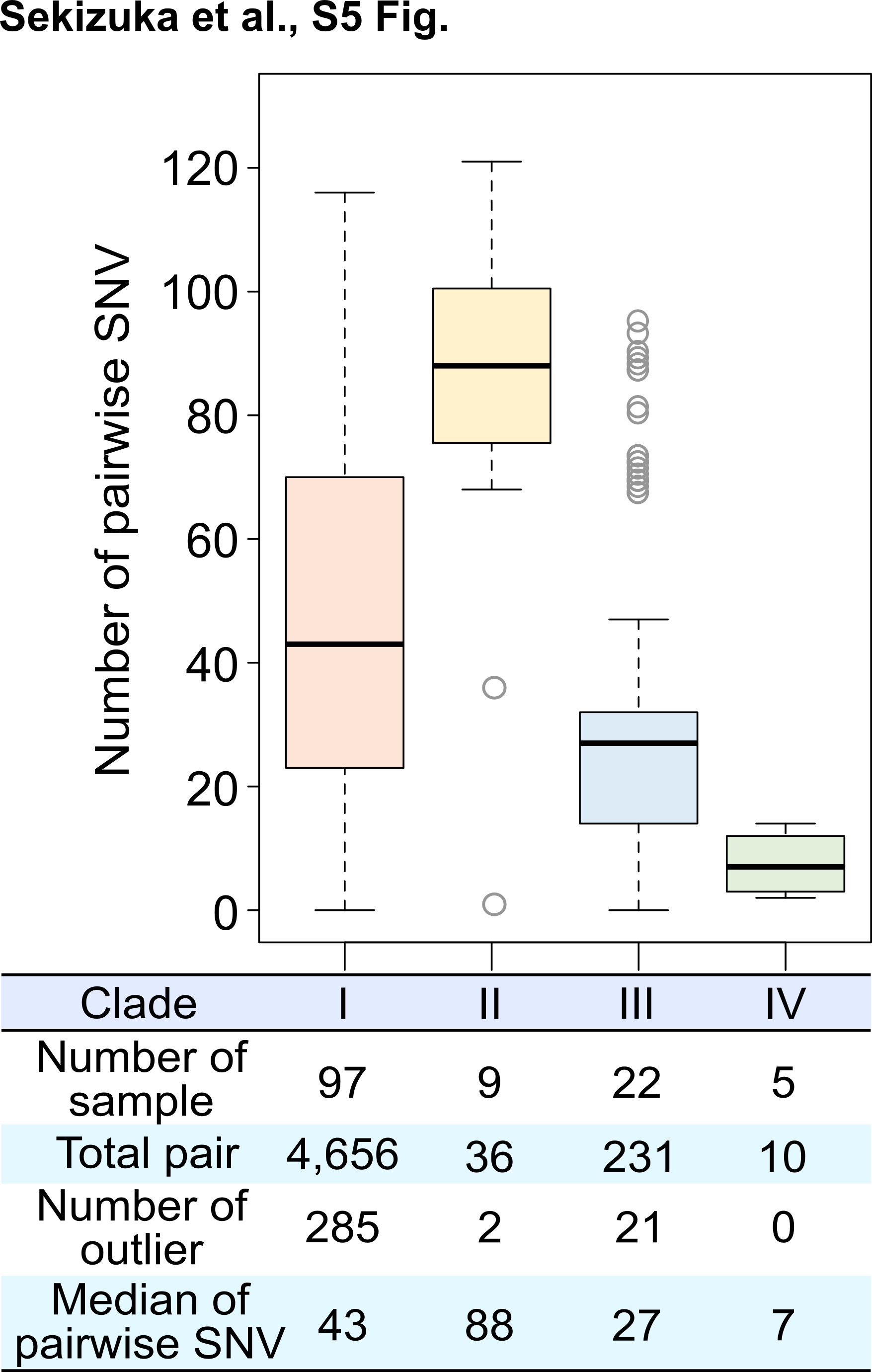

Supplement: S5 Fig — All-against-all comparisons of SNV counts were performed among each clade. Statistical analysis was performed using Wilcoxon rank sum test; the difference was statistically significant (p < 0.001) among all clusters. The boxplot indicates that median of pairwise SNV of clade I, III and IV is lower than those of clade II because of including outbreak case. (TIFF) [file pone.0223433.s009.tiff]

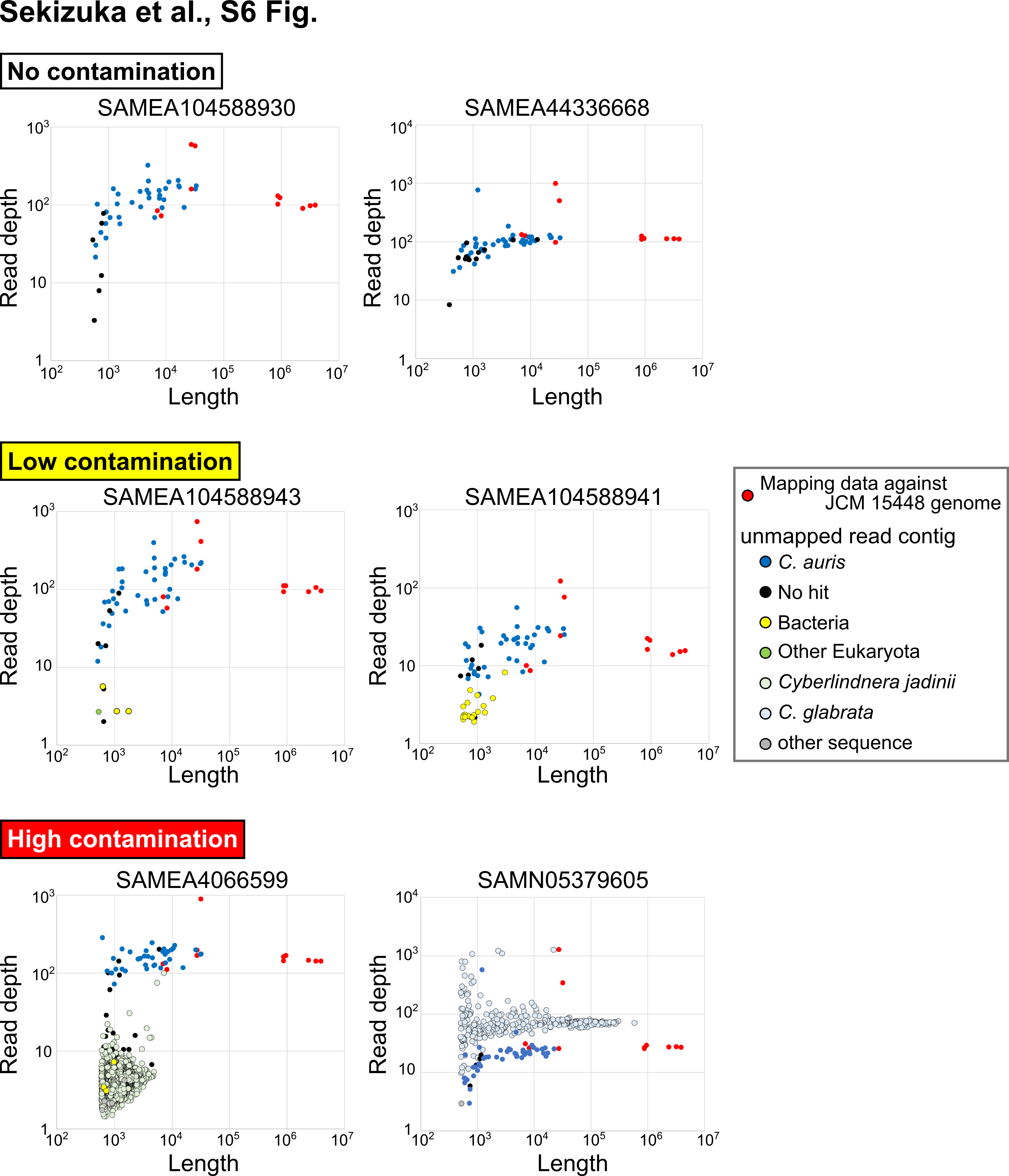

Supplement: S6 Fig — The contigs assembled with unmapped reads against reference sequences were analyzed by BLASTN, followed by taxonomic classification of contigs. These data reveal that the average read depth of non-C. auris contigs show different distributions from those of C. auris contigs. (TIFF) [file pone.0223433.s010.tiff]

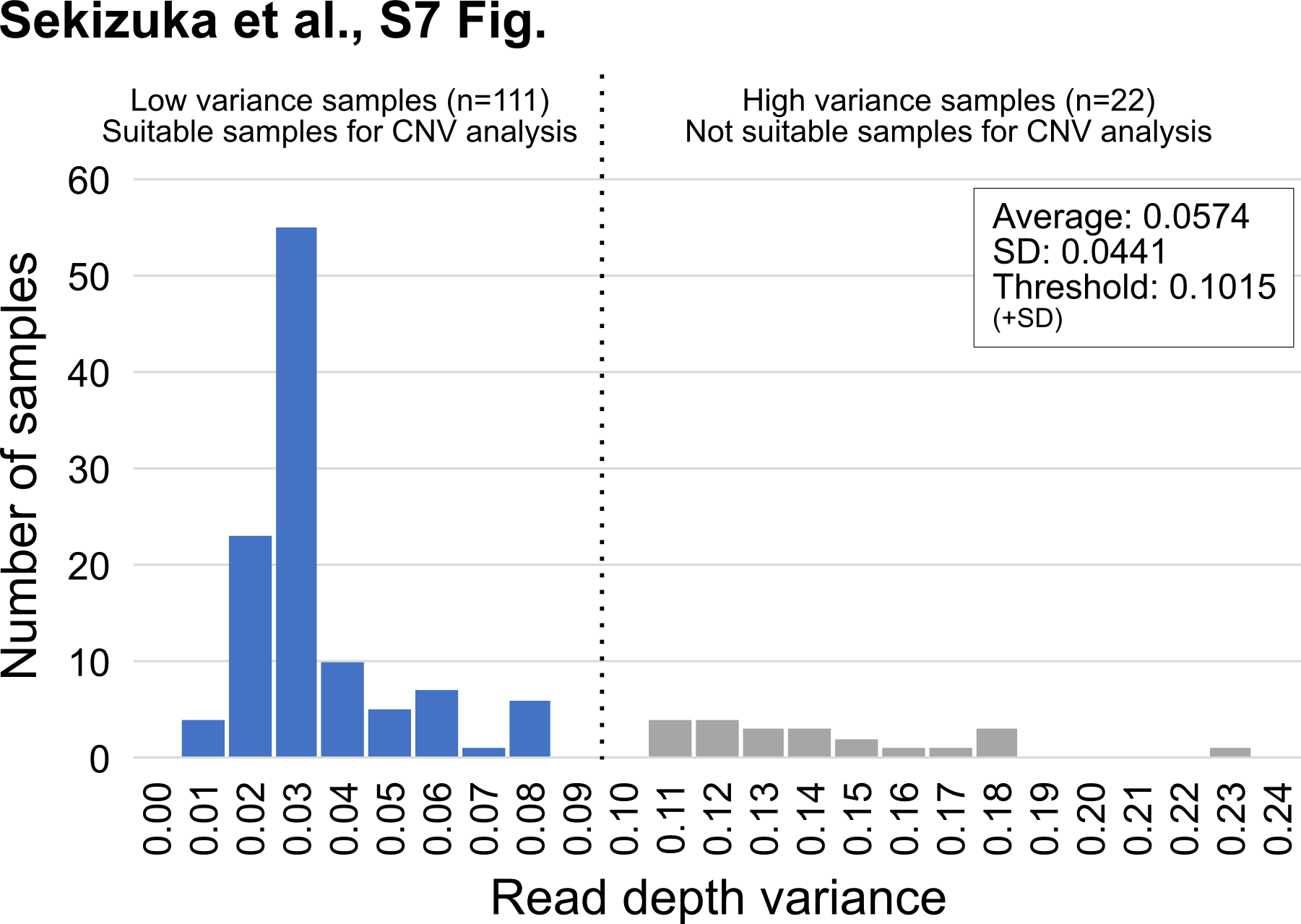

Supplement: S7 Fig — This graph shows a histogram of read mapping variances. Twenty-two samples were rejected in CNV analysis because of abnormal distribution of read mapping depth. These all samples are included in same BioProject ID (PRJEB20230). (TIFF) [file pone.0223433.s011.tiff]

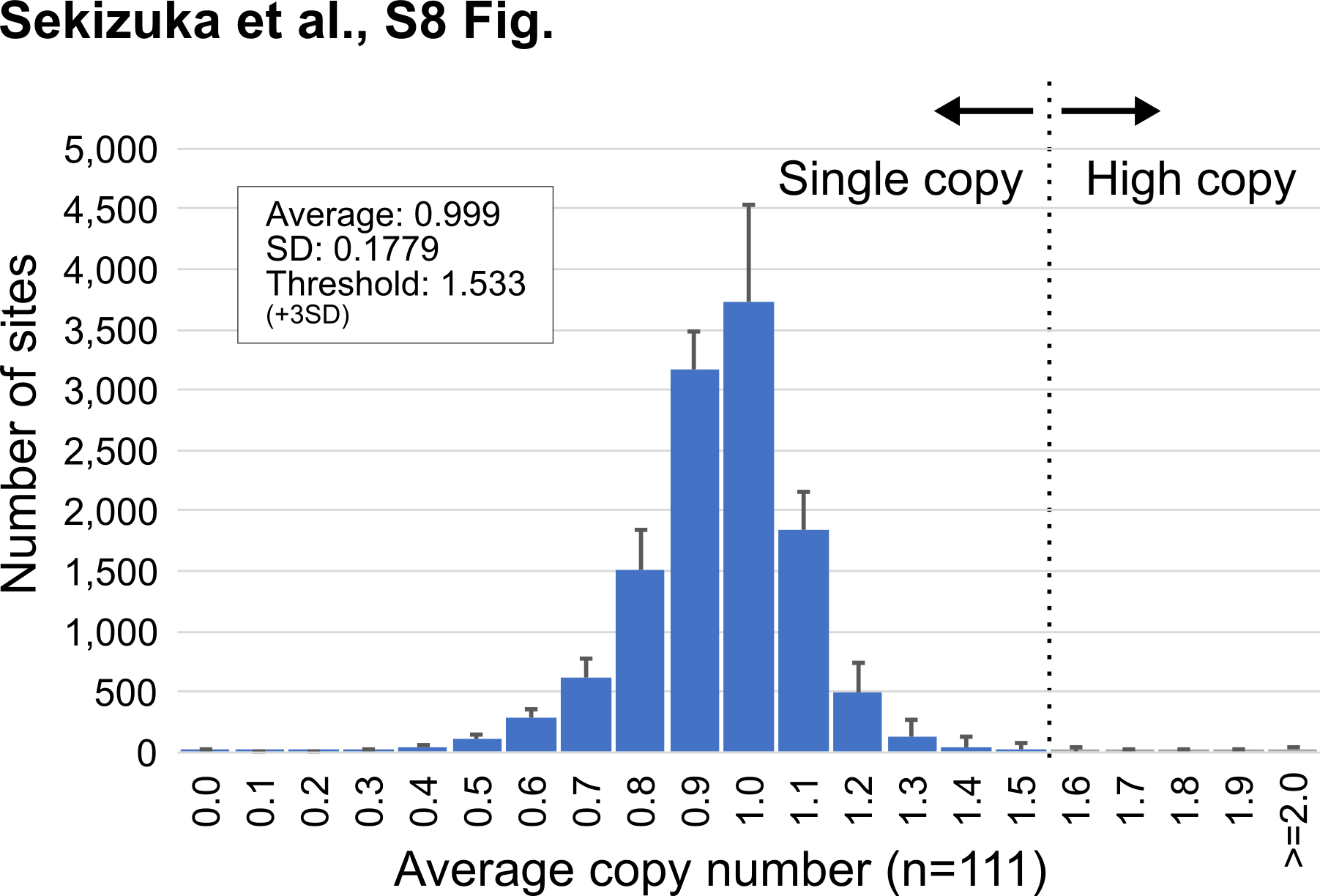

Supplement: S8 Fig — This graph shows a histogram of average copy number in 111 samples which are collected by mapping variance analysis. Error bars represent standard deviation. The statistical data shows that lower than approximately 1.5 and more than 1.6 copy numbers are single and high copy, respectively. (TIFF) [file pone.0223433.s012.tiff]

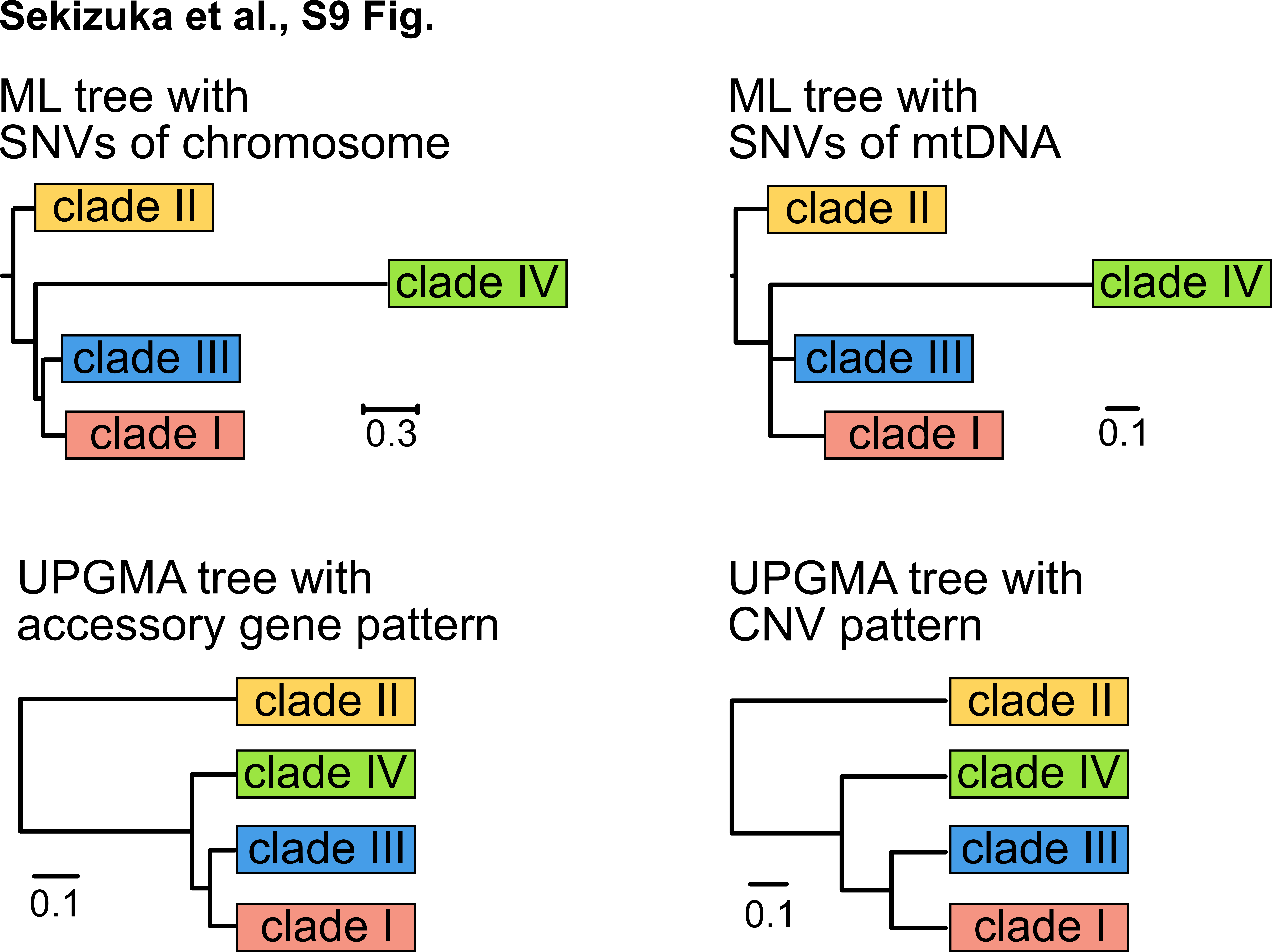

Supplement: S9 Fig — These trees indicate similar topology pattern, and the clade II belongs out of group against clade I, III and IV. (TIFF) [file pone.0223433.s013.tiff]
