# Supplementary material for: Clade II Candida auris possess genomic structural variations related to an ancestral strain
Source: PLoS One. 2019 Oct 9;14(10):e0223433. doi: 10.1371/journal.pone.0223433 (PMC6785063; doi:10.1371/journal.pone.0223433)
Supplement: S1 Table — (PDF) [file pone.0223433.s014.pdf]

S1 Table. Summary of genome assembly statistics of *C. auris* strains.

Strain	Accession number	Clade	Country	Total size (bp)	Scaffolds	Contigs	Scaffold N50 (bp)	GC content (%)	Protein coding genes
JCM 15448	BGOX01000000, AP018713	II	Japan	12,125,386	N/A[a]	12	3,155,023	45.2	5,238
B8441	PEKT00000000	I	Pakistan	12,365,959	15	18	1,083,522	45.2	5,421
B11220	PYFR00000000	II	Japan	12,111,869	320	327	60,224	45	5,546
B11221	PGLS00000000	III	South Africa	12,741,178	20	23	2,363,944	45.3	5,527
B11243	PYGM00000000	IV	Venezuela	12,354,602	238	282	86,716	45	5,601

N/A, not available; [a] There is no gap region with "N" letters.
