# Supplementary material for: Clade II Candida auris possess genomic structural variations related to an ancestral strain
Source: PLoS One. 2019 Oct 9;14(10):e0223433. doi: 10.1371/journal.pone.0223433 (PMC6785063; doi:10.1371/journal.pone.0223433)
Supplement: S2 Table — (PDF) [file pone.0223433.s015.pdf]

S2 Table. Summary of antifungal drug resistance related gene of *C. albicans* and protein homology search with *C. auris* JCM 15448.

			C. albicans		***C. auris* JCM 15448**			
**Function**	**Gene name**	**Inhibitor**	**Accession number (protein)**	**Length**	**Gene number (Locus tag ID)**	**Length**	**Identity (reference: *C. albicans*)**	**Gene position**
Ergosterol biosynthesis pathway	*ERG10*		AOW27457	402	CAJCM15448_03520	400	77.39%	contig_01: 813530..814732
	ERG13		AOW31581	451	CAJCM15448_44470	446	71.69%	contig_04: 874815..876155
	HMG1		AOW26055	1,073	CAJCM15448_08270	1,019	62.29%	contig_01: complement(1895163..1898222)
	ERG12		AOW26579	431	CAJCM15448_51900	425	55.50%	contig_06: 815147..816424
	ERG8		AOW28958	432	CAJCM15448_18590	432	55.76%	contig_02: 447424..448722
	ERG19 (MVD)		AOW25709	362	CAJCM15448_36150	385	71.47%	contig_03: complement(join(1295111..1296223,1296270..1296314))
	IDI1		AOW28999	284	CAJCM15448_18870	281	67.62%	contig_02: 511518..512363
	ERG20		AOW27484	351	CAJCM15448_09210	350	78.51%	contig_01: complement(2101134..2102186)
	ERG9		BAA13995	448	CAJCM15448_43670	445	63.45%	contig_04: 660713..662050
	ERG1	allylamines	AAC49715	496	CAJCM15448_21120	494	66.19%	contig_02: complement(1051410..1052894)
	ERG7		AAA34342	728	CAJCM15448_12350	737	67.67%	contig_01: complement(2811257..2813470)
	ERG11	azoles	CAA31658	528	CAJCM15448_19130	524	70.57%	contig_02: complement(566242..567816)
	ERG24	morpholines	AOW27923	448	CAJCM15448_22310	441	66.74%	contig_02: complement(1309895..1311220)
	ERG25		AAC06014	308	CAJCM15448_20140	303	76.59%	contig_02: join(853136..853143,853206..854109)
	ERG26		AOW29353	350	CAJCM15448_35460	351	72.67%	contig_03: complement(1125892..1126947)
	ERG27		AOW30876	346	CAJCM15448_46890	343	61.40%	contig_05: complement(522931..523962)
	ERG6		AOW28252	376	CAJCM15448_15510	375	86.76%	contig_01: complement(3529375..3530502)
	ERG2	morpholines	AOW25777	217	CAJCM15448_11770	218	66.99%	contig_01: 2679855..2680511
	ERG3	azoles	AAC99343	386	CAJCM15448_03450	363	67.10%	contig_01: 798476..799567
	ERG5		AOW30631	517	CAJCM15448_50480	522	77.41%	contig_06: complement(502071..503639)
	ERG4		AOW28125	469	CAJCM15448_23860	462	69.03%	contig_02: 1654178..1655566
